# Genomic Prediction across Structured Hybrid Populations and Environments in Maize

**DOI:** 10.3390/plants10061174

**Published:** 2021-06-09

**Authors:** Dongdong Li, Zhenxiang Xu, Riliang Gu, Pingxi Wang, Jialiang Xu, Dengxiang Du, Junjie Fu, Jianhua Wang, Hongwei Zhang, Guoying Wang

**Affiliations:** 1 National Key Facility for Crop Gene Resources and Genetic Improvement, Institute of Crop Sciences, Chinese Academy of Agricultural Sciences, Beijing 100081, China; 15993216250@163.com (D.L.); wangpingxi2009@163.com (P.W.); xujialiang2017@163.com (J.X.); fujunjie@caas.cn (J.F.); 2 Center for Seed Science and Technology, College of Agronomy and Biotechnology, China Agricultural University, Beijing 100193, China; dlxuzhenxiang@163.com (Z.X.); 06026@cau.edu.cn (R.G.); wangjh63@cau.edu.cn (J.W.); 3 National Key Laboratory of Crop Genetic Improvement, Huazhong Agricultural University, Wuhan 430070, China; ddx@mail.hzau.edu.cn

**Keywords:** maize, genomic prediction, genotype by environment, hybrid prediction, yield per plant

## Abstract

Genomic prediction (GP) across different populations and environments should be enhanced to increase the efficiency of crop breeding. In this study, four populations were constructed and genotyped with DNA chips containing 55,000 SNPs. These populations were testcrossed to a common tester, generating four hybrid populations. Yields of the four hybrid populations were evaluated in three environments. We demonstrated by using real data that the prediction accuracies of GP across structured hybrid populations were lower than those of within-population GP. Including relatives of the validation population in the training population could increase the prediction accuracies of GP across structured hybrid populations drastically. G × E models (including main and genotype-by-environment effect) had better performance than single environment (within environment) and across environment (including only main effect) GP models in the structured hybrid population, especially in the environment where yields had higher heritability. GP by implementing G × E models in two cross-validation schemes indicated that, to increase the prediction accuracy of a new hybrid line, it would be better to field-test the hybrid line in at least one environment. Our results would be helpful for designing training population and planning field testing in hybrid breeding.

## 1. Introduction

Global climate change and population growth are impending threats to food security; crop varieties with wide adaptability and high yield should be developed to feed the growing population [1]. Rich genetic resources are the basic materials for breeding new varieties, and the favorable alleles from genetic resources should be introgressed into elite inbred lines or varieties [2,3]. Genomic prediction (GP) can predict the performance of crop germplasm, and can be used to accelerate the introgression [4]. Compared with other molecular breeding tools such as marker-assisted selection and marker-assisted recurrent selection, GP simultaneously estimates the marker effects and computes breeding values and removes the need for testing marker–trait association and selecting significant marker/QTL (quantitative trait locus/loci). Moreover, GP can increase breeding efficiency by accelerating the breeding cycle and enhancing annual genetic gain [5]. Therefore, GP shows great prospects in commercial crop breeding in the context of continuing population growth.

In GP, two populations need to be established: the training population with both phenotypic and genotypic data, and the validation population with only genotypic data. The phenotype of the validation population can be predicted once phenotypic effects of genome-wide molecular markers are estimated in the training population. Generally, the prediction accuracy (PA) of GP was defined as the correlation between the predicted and observed phenotype of the validation population. The PA of GP was related to many factors including training population size, number of markers, heritability, and the relationship between training and validation populations [6,7,8]. The low PA of GP across different populations reduced the selection efficiency and had become a major obstacle for implementing GP in crop breeding [7,9]. Although several studies had examined the rationales of the low PAs of GP across different populations [7,10], few efficient methods were proposed on how to solve this problem.

Compared to the prediction of inbred line performance, predicting hybrid performance has practical significance in crop breeding. If selection can be made on the basis of predicted hybrid performance, hybrid seeds production and field evaluation will be unnecessary for a majority of hybrid lines, especially when the field performance of an extremely large number of testcrosses or hybrids needs to be evaluated. For example, instead of evaluating the performance of 21,945 potential hybrids that could be developed by crossing 210 recombinant inbred lines, Xu et al. [11] used 278 hybrids as the training population and predicted the performance of the 21,945 potential hybrids. In another report, the population containing 1495 hybrids from a public resource was used as the training population [12], the performance of 44,636 potential hybrids that could be developed through using 3000 germplasm accessions were predicted, and 200 best potential hybrids were identified on the basis of predicted performance [13,14]. Generally, hybrid prediction would save the investments for developing and evaluating hybrid lines and increase the efficiency of breeding hybrid varieties.

QTL controlling crop traits (such as yield-related traits and flowering time) showed significant QTL × environmental interaction [15,16], indicating that field performances of crop plants were not only related to their genetic basis, but also to their interactions with environments. As GP was related to the genetic basis of the target trait and the genetic basis interacted with environments, GP should be related to the interactions between genotypes and environments (G × E) [17]. Previous reports found that including G × E effects in GP models (G × E models) increased PAs in crop breeding populations [18,19], but the performances of G × E models were rarely assessed in structured hybrid populations.

In order to find solutions to increase PA of GP across structured hybrid populations and environments, we constructed four maize hybrid populations and evaluated yields of these populations. We firstly proved that the four populations shared different levels of genetic relatedness. Then, we designed different GP schemes to find a better way to increase PA of GP across structured hybrid populations. Through implementing three multi-environment models in two CV (cross-validation) schemes, we found that G × E models had the best performance in the structured hybrid population, and there were differences between PAs of the two CV schemes.

## 2. Results

### 2.1. Phenotypic Data Analysis

Among the three environments, yield was largest in XJ (Xinjiang), followed by BJ (Beijing) and HN (Henan) (Figure S1a). Among the four hybrid populations, population 2 had the highest yield, followed by population 1, 4 and 3, respectively, suggesting that population 2 had the best special combining ability with the tester PH6WC (Table 1; Figure S1b). Population 4 had the largest coefficient of variance, followed by population 2, 1 and 3, suggesting that population 4 had the largest phenotypic variation (Table 1). The large coefficient of variance of population 4 might be related to the fact that the parental lines of population 4 were breeding materials, whereas those of other populations were biparental populations. For each population, the correlation among the three environments was mostly significant, suggesting that the genetic basis played a major role across the three environments (Table S1). For each population, ANOVA (analysis of variance) revealed that the genotypic variation was significant. The linear model fitted well for each population, the determination coefficients ($R^{2}$) were 91.24%, 86.05%, 84.52%, and 88.94%, respectively (Table 2). *H*^2^ (entry-mean broad sense heritability) across environments ranged from 0.58 for population 3 to 0.73 for population 4. Meanwhile, *H*^2^ values of the four populations (as a whole) were 0.63, 0.68 and 0.59 in BJ, XJ and HN environments, respectively, and the final heritability of whole population across three environments was 0.72 (Table S2), suggesting yield was stable across environments. Generally, the genotypic variation was significant and genetic factors played an important role in determining yield across environments.

### 2.2. The Genetic Relationships of Multiple Populations

**Figure 1:**
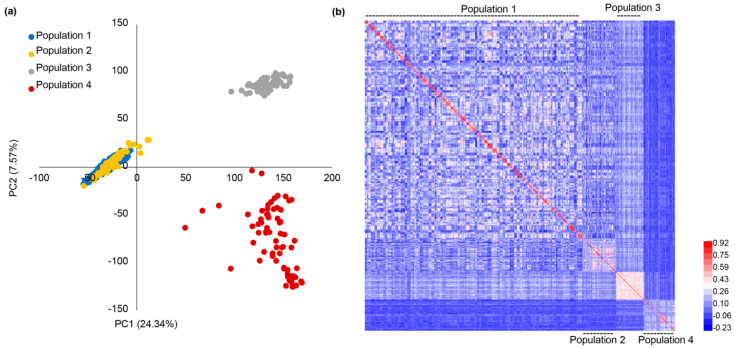
PC analysis and genetic similarity among the four populations. (**a**) PC analysis of the four populations on the basis of 18,702 SNP markers; (**b**) the genetic similarity heatmap was used to demonstrate the genetic relatedness among the four populations.

In total, 18,702 high-quality SNPs were obtained after SNP filtering and imputation. These SNPs were distributed evenly across the ten chromosomes (Figure S2), and the marker density was sufficiently high for implementing GP [8]. Considering there were pedigree relationships among some populations, the genotypic data were used to show the genetic relationships among the four populations. PC (principal component) analysis showed that PC1 and PC2 explained 24.34% and 7.57% of total variances (Figure 1a). The two PCs could divide the four populations into three groups, with population 1 and 2 in one group, and population 3 and 4 in another two groups (Figure 1a). The genetic similarity matrix was used to plot the heatmap, which showed that relationships among population 1, population 2, and population 3 were close. The relationship between population 4 and other populations was distant (Figure 1b). The genetic similarity matrix generally complied with their relationships in that population 1 and 2 had two common parents, and population 3 had one common parent with population 1 and 2.

### 2.3. GP across Different Populations

**Figure 2:**
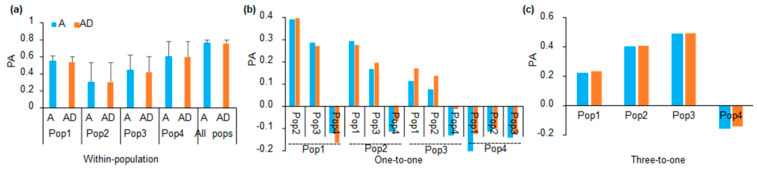
GP across different populations. (**a**) PA of the within-population GP, which was performed with five-fold CV and was repeated 100 times. A and AD were the GBLUP models including only additive effect, and additive plus dominance effects, respectively; Pop1-Pop4 were population 1–4, and all pops indicated all populations were used together to perform within-population GP (**b**) PA of the one-to-one prediction scheme. The lower name in the x-axis is the training population, and the upper name in the x-axis is the validation population; (**c**) PA of the three-to-one prediction scheme. The populations in the x-axis are the validation population, and the remaining three populations were used together as the training population.

For within-population GP, the PA of GP within population 4 was higher than that of GP within population 1, 2, and 3. PA was the highest when the four populations were combined as a whole population to perform within-population GP (Figure 2a). The large standard deviations of PAs of within-population GP for population 2 to 4 might be caused by their small population size. For within-population GP of each population, no significant differences were observed between the PAs of A (additive) and AD (additive and dominance) models (Figure 2a). For the one-to-one GP, when population 1 was used as the training population and each of the other three populations were used as the validation population, the ranking PAs from high to low were population 2, population 3 and population 4 (as the validation populations) (Figure 2b), respectively. When population 2 was used as the training population, the PA was largest when population 1 was used as the validation population and lowest for when population 4 was used as the validation population (Figure 2b). When population 3 was used as the training population, the PA was largest when population 1 was used as the validation population and lowest when population 4 was used as the validation population (Figure 2b). When population 4 was used as the training population, the PA was negative when each of the remaining populations were used as the validation population (Figure 2b). Generally, the PAs of one-to-one GP models complied with their genetic relatedness.

We performed GP through using the three-to-one scheme, in which one population was used as the validation population, and the other populations were combined as the training population. The result showed the PAs of GP using population 3 as the validation population were the largest, followed by the PAs of GP using population 2, 1 and 4 as the validation populations, respectively (Figure 2c). We further compared the PAs of the three-to-one schemes with those of the one-to-one schemes. When population 1–3 was used as the validation population, the PAs of the three-to-one scheme were generally larger than those of the one-to-one scheme (Figure 2c). When population 4 was used as the validation population, the PA of the three-to-one scheme was as low as the PAs of one-to-one GP models (Figure 2c). The comparison indicated that the PA of GP using population 4 as the validation population was low in both GP schemes (one-to-one and three-to-one), which might be related to the fact that population 4 was genetically unrelated to other populations (Figure 1a,b). Furthermore, because population 1–3 shared comment parents, the comparison also indicated that including relatives of the validation population in the training population increased the PAs of GP across structured populations.

### 2.4. GP across Different Environments

**Figure 3:**
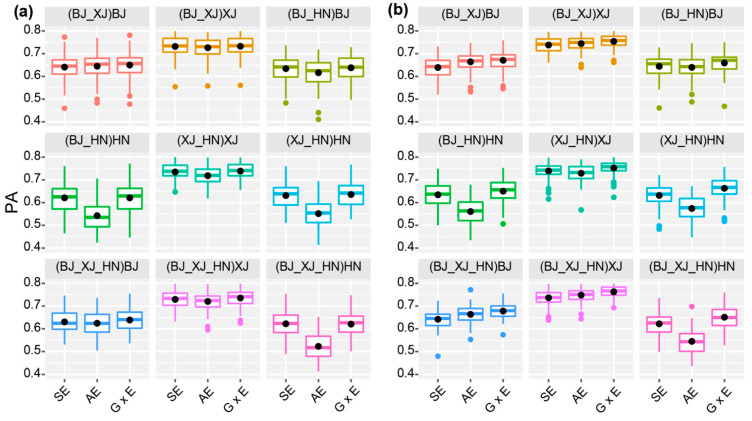
PA of the SE, AE, and G × E models for CV1 (**a**) and CV2 (**b**), respectively. The data inside and outside of brackets are the training, and validation datasets, respectively.

To compare the PAs of GP across environments, we implemented three models (SE (single environment model), AE (across environment model), and G × E models) by using the four hybrid populations as a whole in two CV schemes (Table S3). For CV1, the PAs ranged from 0.522 in AE model (when 20% lines in HN environment were treated as the validation population and the other 80% of lines in all three environments were treated as the training population) to 0.744 (when 20% of lines in XJ environment were treated as the validation population and the other 80% of lines in both XJ and HN environments were treated as the training population) in the G × E model (Table S4). G × E models generally had higher PAs than SE and AE models (Figure 3a, Table S4). For CV2, the PAs ranged from 0.543 in AE model (when 20% of lines in HN environment were treated as the validation population and 80% of lines in all three environments were treated as the training population) to 0.769 (when 20% lines in XJ environment were treated as the validation population and 80% of lines in all three environments were treated as the training population) in the G × E model. In all cases, the G × E model had the highest PA in the CV2 scheme (Figure 3a,b, Table S4). These results indicated that the G × E model had the best performance in both the CV1 and CV2 schemes.

We also found that the PAs were always the highest when lines in BJ were used as the validation population, and lowest when lines in HN were used as the validation population for all models (Figure 3a,b, Table S4), which should be related to the fact that yield in XJ had the highest heritability, and that in HN it had the lowest heritability (Table S2). In addition, for both AE and G × E models, the PAs of the CV2 scheme were always higher than those of the CV1 scheme. In the CV2 scheme, each line was evaluated in at least one environment, suggesting that GP worked better for lines with phenotypic data in some environments.

## 3. Discussion

GP had been proved useful for maize breeding, especially for complex traits (such as yield and stress tolerance) [20,21]. Although research on GP on the basis of multiple populations had been reported in plants and animals before [7,9,22], the populations used in these published reports were natural populations or populations composed of homozygous lines (such as DH (doubled haploid) populations). Although DH lines were widely used in commercial maize breeding, second-cycle lines and backcross lines were still used extensively in developing countries. Moreover, in practical breeding, breeders would like to make decisions at different generations on the basis of field testing. Therefore, investigating GP in multiple types of population (such as backcross populations and F_4_ population in this study) should have great significance. Our result demonstrated that GP was reliable in DH, backcross, F_4_ populations, which would encourage early testing in hybrid breeding in developing countries.

We showed that the PA of GP across different populations was related to the relationship between the training and validation populations (Figure 2). For example, because populations 1–3 shared one common parent, when population 1 was used as the training population in the one-to-one scheme, the PA of GP was highest when population 2 was used as the validation population, and lowest PA when population 4 was used as the validation population (Figure 2b). Therefore, genetic relatedness between the training and validation populations was an important factor causing variations of the PAs of GP. Furthermore, the differences in genetic basis of yield among the four populations might be another important factor influencing the PAs of GP [10]. If the training and validation populations were unrelated, their genetic basis should be different, and the effective SNPs detected in the training population might have no effects in validation population. Therefore, the closer the genetic relationship between the training and validation populations was, the more genetic basis they would have in common, leading to the consequence that the number of SNPs taking effects in both populations would increase, and the PAs of GP across different populations would also increase. Based on this speculation, we assumed that when the relatives of the validation population were included in the training population, the overall genetic relationship between the training and validation populations would be close. Therefore, we designed the three-to-one scheme to test this assumption. Comparison between the PAs of the three-to-one and one-to-one schemes supported our speculation and indicated that including relatives of the validation in the training population was a good choice for increasing the PAs of GP across structured populations. Conclusively, the increase in PA might be caused by the possibility that, when the individuals of the validation population were included in the training population, the common genetic basis between the training population and validation populations would increase.

The conclusion that including relatives of the validation population in the training population could enhance GP will be helpful for the design of the training population. For GP, if breeders wanted to select favorable lines from the offsprings of two lines, a training population should be constructed through using the two lines [23]. In most cases, there might be several GP projects in a company. For n projects (n is an integer ≥ 2), n populations needed to be constructed by using 2 × n parents (Figure S3). There might be two cases: (1) some of the 2 × n parents may be common; (2) some parents may be genetically related. In both cases, pooling the training populations as one training population would enhance GP, because many relative lines of the validation populations were included in the training population. Therefore, for GP on the basis of multiple populations, we suggested combining multiple training populations as one training population, especially when the parents used in different GP projects were common or genetically related.

We used CV1 and CV2 to compare the multi-environment GP models, finding that the PAs of CV2 were larger than those of CV1 in most cases (Figure 3; Table S4). The result indicated that, to accurately predict the performance of an untested hybrid line in a specific environment, it would be better to evaluate this line in at least one environment. The multi-environment GP also found that G × E models had better performance than the SE and AE models, especially in environments where the yield had higher heritability. Our finding indicated that G × E models should be used to predict hybrid performance in structured populations, and that the PAs of G × E models in a specific environment were related to the heritability of the traits tested in the environment.

## 4. Material and Methods

### 4.1. Plant Materials

Through using Zheng58 as the donor parent and PH4CV as the recurrent parent, we produced BC_1_F_1_ in the winter of 2013 in Sanya, Hainan province. In the summer of 2014, BC_1_F_1_ were sown in Shunyi, Beijing. We used pollens bulking from at least ten BC_1_F_1_ plants to pollinate other BC_1_F_1_ plants because bulked-pollen pollination could increase the proportion of genome from the recurrent parent in the backcross population [24]. The seeds of these BC_1_F_1_ plants were called bulk-BC_1_F_2_. Bulk-BC_1_F_2_ seeds were sown and self-pollinated to produce BC_1_F_3_ seeds in the summer of 2015 in Shunyi, Beijing [19]. In the winter of 2015 in Sanya, Hainan province, 475 BC_1_F_3_ plants were testcrossed to PH6WC, and this testcross population was called population 1. Here, PH4CV and PH6WC were the male and female parents of Xianyu335, Zheng58 was the female parent of Zhengdan958. Xianyu335 and Zhengdan958 were popular hybrid varieties in China [25,26]. The bulk-BC_1_F_2_ seeds were used to generate 72 DH lines, which were testcrossed to PH6WC, and this testcross population was called population 2. Zheng58 was crossed to the inbred line D1-sel to produce F_1_ seeds, which were used to produce 60 F_4:5_ families through continual self-pollination. These 60 families were testcrossed to PH6WC, and the testcross population was called population 3. Here, the inbred line D1-sel was selected from the offsprings of Demeiya 1, which was a popular hybrid variety in northeast China. Sixty-eight breeding materials (Table S5), most of which were homozygous lines, were testcrossed to PH6WC, and the testcross population was called population 4. A diagram illustrating how to develop these four populations is shown in Figure S4.

### 4.2. Phenotype Evaluation and Phenotypic Data Analysis

In the summer of 2017, the four hybrid populations were sown in Shunyi (Beijing Municipality, N 40°07′, E 116°39′), Xinxiang (Henan province, N 35°22′, E 113°54′) and Changji (Xinjiang Uygur Autonomous Region, N 44°0′, E 87°18′). The whole population was arranged in an incomplete block design with two replications. Specifically, because populations 2, 3, and 4 were small populations, each of these populations was in one block. As population 1 contained 475 families, population 1 was divided into eight random blocks with five blocks containing 59 lines, and three blocks containing 60 lines. Each hybrid line was sown in a two-row plot, and the row length, row space, and inter-plant space in the same row were 500, 60, and 25 cm, respectively. The planting density was approximately 4444 plants per mu (mu is used widely in China), and one mu was equal to 666.67 square meters. The management in the field followed the normal agricultural practice [27]. At the harvest stage, the yield of each plot was measured and adjusted to 14% water content. Yield (yield per mu) was calculated on the basis of the yield per plot. The three environments were called BJ (Beijing), HN (Henan) and XJ (Xinjiang), respectively.

Best linear unbiased estimations (BLUEs) of each population were estimated based on the experimental design following the model:

(1) $$y_{ijk}=\mu +g_{i}+e_{j}+ge_{ij}+\delta_{\left(j\right)k}+\varepsilon_{ijk}$$

where $y_{ijk}$ is the yield of $i^{th}$ genotype in the $k^{th}$ (*k* = 1,2) replicate nested in the $j^{th}$ (*j* = 1,2,3) environment, μ is the overall mean, $g_{i}$ is the genotype effect, $e_{j}$ is the environment effect, $ge_{ij}$ is G × E effect, $\delta_{\left(j\right)k}$ is the replicate effect nested in each environment, and $\varepsilon_{ijk}$ is the residual error. To calculate BLUEs, genotype was treated as a fixed effect, and the other factors were treated as random effects following normal distributions. The linear mixed model was fitted through using the lmer function in the R package “lme4” [28]. The BLUEs of the four testcross population were available as Supplementary File S1.

To calculate the broad-sense heritability ($H^{2}$), the variance of each effect in model (1) were dissected by using the ANOVA function in software IciMapping version 4.1 [29]. This time, all effects in model (1) were treated as random effects. The equation for estimating $H^{2}$ was [30]:

(2) $$H^{2}=\frac{\sigma_{g}^{2}}{\sigma_{g}^{2}+\frac{\sigma_{ge}^{2}}{e}+\frac{\sigma_{\varepsilon }^{2}}{er}},$$

where $\sigma_{g}^{2}$ is the genotype variance, $\sigma_{ge}^{2}$ is the variance of G × E, $\sigma_{\varepsilon }^{2}$ is the error variance, and *e* and *r* were the number of environments and replicates, respectively.

### 4.3. Genotyping and Genotypic Data Analysis

Leaves were collected from the populations, and DNA was extracted according to a published protocol [31]. The DNA samples were sent to CapitalBio Corporation for genotyping by using DNA chips containing 55,000 SNPs [32]. The raw genotype data was filtered by using the following requirement: (1) SNPs with calling rate lower than 97% were removed; (2) SNPs with no physical position information were removed; (3) SNPs with missing rate larger than 1% were removed; (4) SNPs with minor allele frequencies lower than 0.05 were removed; (5) the missing genotypes were imputed by using codeGen function in the R package “synbreed”, the method “beagle” was used and the other settings were default [33]. The minor and major alleles were coded as “A” and “a”, respectively [34]. The genotype codes of each hybrid line were inferred from their parents according to a previous report [14]. Briefly, the additive genotypes of hybrid lines were coded as (0 + 1)/2 = 0.5 when the mating type for a locus was Aa × AA, and were coded as (0 + (−1))/2 = −0.5 when the mating type was Aa × aa. For the dominance genotypes of the hybrid line, both above-mentioned mating types were coded as 0.5. The additive and dominance genotype codes were available as Supplementary File S2 and Supplementary File S3, respectively.

PC analysis was performed by using 18,702 filtered SNPs following a previous report [35]. Briefly, the genotype matrix was decomposed by using the svd (singular value decomposition) function in R language, and the matrix whose columns contained the right singular vectors was returned. PC was calculated by multiplying the scaled genotype matrix by the returned matrix. The genetic similarity matrix among all lines was calculated by using the Pearson correlation coefficients among the genotype matrix of all lines [9].

### 4.4. GP Model

BLUE values of the four hybrid populations were used to implement GS. A genomic BLUP (GBLUP) model including additive and dominance genetic effects could be written as [36]:

(3) $$y=\mu +\xi_{a}+\xi_{d}+\varepsilon$$

where ***y*** is the BLUE of F_1_ hybrid, μ is the overall mean, $\xi_{a}$ is the vector of additive polygenic effect following the distribution $\xi_{a}$~N(0, $K_{a}\sigma_{a}^{2}$), $\xi_{d}$ is the vector of dominance polygenic effect following the distribution $\xi_{d}$~N(0, $K_{d}\sigma_{d}^{2}$), and ε is the residual with a normal distribution $\varepsilon \sim N\left(0,\;I\sigma_{\varepsilon }^{2}\right)$, where I is the identity matrix, and $\sigma_{\varepsilon }^{2}$ is the residual variance. The design matrices of $\xi_{a}$ and $\xi_{d}$ are identity matrices. The kinship matrix of additive polygenic effect $K_{a}$ $=K_{a}^{*}/\mathrm{mean}\left(\mathrm{diag}\left(K_{a}^{*}\right)\right)$, where $K_{a}^{*}$= $ZZ^{T}$; the kinship matrix of dominance polygenic effect = $K_{d}$/mean(diag($K_{d}^{*}$)), where $K_{d}^{*}$ = $WW^{T}$ [36]. Z and W are the additive and dominance genotype matrices, respectively, and were coded following a former study [14]. The linear mixed model was fitted by using a Bayesian Reproducing Kernel Hilbert Spaces Regressions regression in BGLR package [37] (the parameter nIter and burnin were 15,000 and 1000, respectively). The model only including additive effect was named the A model, and the model including both additive and dominance effects was named the AD model.

### 4.5. GP across Environments

Three GP models including the SE, AE and G × E models were implemented [18,19], and only additive effect was included in these models. The phenotypic data (response variable) were BLUE values calculated in each environment.

In the SE model, we used model (3) to implement GP with 100 five-fold CVs, where dominance effects were excluded from the model. The response variable ***y*** was the BLUE values in each environment. For the AE model, all marker effects were assumed to be constant under all environments, so the genetic effect was also constant across all environments, taking a pair of environments as an example, the GBLUP model was:

(4) $$\left(\begin{array}{c} y_{1}\\ y_{2} \end{array}\right)=\left(\begin{array}{c} 1\mu_{1}\\ 1\mu_{2} \end{array}\right)+\left(\begin{array}{c} \xi_{1}\\ \xi_{2} \end{array}\right)+\left(\begin{array}{c} \varepsilon_{1}\\ \varepsilon_{2} \end{array}\right)$$

and could be written as:

(5) y=μ+ξ+ε

where $y_{1}$ and $y_{2}$ are the BLUE values in each environment; $\mu_{1}$ and $\mu_{2}$ are the overall means in each environment; $\xi_{1}$ and $\xi_{2}$ re the genetic effect containing only the additive effect; $\varepsilon_{1}$ and $\varepsilon_{2}$ are the error term in each environment, where ($\xi_{1}$, $\xi_{2}$)~N(0, $G_{0}\sigma_{u}^{2}$), and $(\varepsilon_{1}$, $\varepsilon_{2}$)~N(0, ***I***$\sigma_{\varepsilon }^{2}$), $\varepsilon_{1}$ and $\varepsilon_{2}$ are assumed to be homogeneous across different environments.

For the G × E model, genetic effect was divided into two parts, one part was constant across environments, and the other was environment specific G × E effect [18]. Taking two environments as an example, the GBLUP model was:

(6) $$\left(\begin{array}{c} y_{1}\\ y_{2} \end{array}\right)=\left(\begin{array}{c} 1\mu_{1}\\ 1\mu_{2} \end{array}\right)+\left(\begin{array}{c} \xi_{1}\\ \xi_{2} \end{array}\right)+\left(\begin{array}{c} \xi_{1s}\\ \xi_{2s} \end{array}\right)+\left(\begin{array}{c} \varepsilon_{1}\\ \varepsilon_{2} \end{array}\right)$$

and could be written as:

(7) $$y=\mu +\xi +\xi_{s}+\varepsilon$$

where $y_{1}$, $y_{2}$, $\xi_{1}$ and $\xi_{2}$ are same as those of the AE model; $\xi_{1s}$ and $\xi_{2s}$ are the G × E effect in two environments, respectively. ($\xi_{1}$, $\xi_{2}$)~N(0, $G_{0}\sigma_{u}^{2}$), ($\xi_{1s}$, $\xi_{2s}$)~N(0, ***G1***), $(\varepsilon_{1}$, $\varepsilon_{2}$)

~N(0, ***I***$\sigma_{\varepsilon }^{2}$), *G*_0_ is the same with the AE model, $\varepsilon_{1}$ and $\varepsilon_{2}$ are assumed to be homogeneous across all environments. For covariance matrices ***G*_0_** and ***G*_1_**, more details can be found in former research [18]. All the models were fitted in BGLR package by providing corresponding covariance matrix ***G*_0_** and ***G*_1_**, respectively.

For the AE and G × E models, GP were implemented using BLUE values across two (or three) environments as the response variable (Table S4) [38]. The model was run with 100 five-fold CVs, with an 80% lines training population and 20% validation population (Table S3). Two CV schemes (CV1 and CV2) were used for the AE and G × E models. In CV1, the phenotype data of some lines were deleted in all environments and were predicted by using the AE and G × E models. For CV2, the phenotype data of some lines were deleted in only one environment and were predicted by using the AE and G × E models. PA was the correlation coefficient of the predicted and observed phenotype data in each environment.

### 4.6. GP across Different Populations

We used three GP schemes, named as within-population, one-to-one, and three-to-one schemes. For the within-population GP scheme, GP was implemented by using BLUE values in each population, 80% lines of each population were used as the training population, and the left 20% lines were used as the validation population (Figure S5a); the GP model was run with 100 five-fold CVs. For the one-to-one GP scheme, each population was used as the training population to predict each of the remaining populations (Figure S5b). For the three-to-one GP scheme, three populations were used together as the training population, and the remaining one was used as the validation population (Figure S5c). PA was the correlation between the predicted and observed phenotype value.

## Figures and Tables

**Table 1:** Summary of basic statistics of yields of the four populations.

Population	N	Mean (Kg)	SD (Kg)	Minimum (Kg)	Maximum (Kg)	Range (Kg)	CV (%)
Population 1	475	753.71	43.55	641.95	947.32	305.37	5.78
Population 2	72	815.52	54.11	691.36	959.49	268.12	6.63
Population 3	60	656.97	34.87	587.86	745.70	157.83	5.31
Population 4	68	687.40	58.63	539.14	840.69	301.55	8.53

Note: N is the population size, SD is standard deviation, and CV is coefficient of variation. Population 1 to population 4 are introduced in detail in the Materials and Methods.

**Table 2:** Variance dissection of yield of the four hybrid populations.

Population	ANOVA						*H* ^2^	*R*^2^ (%)
	Source	DF	SS	MS	F	*p*		
Population 1	Rep/Env	3	122,714.40	40,904.80	11.24	0.00	0.63	91.17
	Genotype	474	5,459,187.50	11,517.27	3.17	0.00		
	Environment	2	39,391,468.00	19,695,734.00	5413.62	0.00		
	G by E	935	4,392,113.50	4697.45	1.29	0.00		
	Error	1314	4,780,569.00	3638.18				
Population 2	Rep/Env	3	24,789.26	8263.09	2.23	0.09	0.66	85.47
	Genotype	71	957,095.81	13,480.22	3.63	0.00		
	Environment	2	2,448,691.25	1,224,345.63	329.97	0.00		
	G by E	130	651,743.31	5013.41	1.35	0.03		
	Error	187	693,851.19	3710.43				
Population 3	Rep/Env	3	3349.01	1116.34	0.34	0.80	0.62	84.52
	Genotype	59	548,701.06	9300.02	2.83	0.00		
	Environment	2	1,797,173.38	898,586.69	273.06	0.00		
	GE_interaction	118	431,993.81	3660.96	1.11	0.27		
	Error	156	513,373.16	3290.85				
Population 4	Rep/Env	3	15,394.80	5131.60	1.56	0.20	0.74	88.79
	Genotype	67	1,310,953.50	19,566.47	5.95	0.00		
	Environment	2	2,757,321.25	1,378,660.63	419.16	0.00		
	GE_interaction	133	762,263.31	5731.30	1.74	0.00		
	Error	186	611,768.25	3289.08				

Note: DF is degree of freedom; SS is sum of squares; MS is mean square of variance; *F* is *F* value of *F*-test; *p* is *p* value of *F*-test; $H^{2}$ is broad-sense heritability; $R^{2}$ is the multiple R-square of fitted linear model. Rep/Env is the replicate effect nested in each environment.

## Data Availability

The data are provided as Supplementary Files S1, S2 and S3.

## Supplementary Materials

The following are available online at https://www.mdpi.com/article/10.3390/plants10061174/s1. Figure S1. Distribution of yield of each population in each environment. (a) Distribution of yield of each population in each environment; BJ, XJ and HN indicate the three environments; (b) Distribution of yield of each population; Figure S2. Density distribution of 18702 SNPs within 1-Mb window size. The marker physical position is based on the B73 RefGen_V3 sequence. Chr1, Chr2…Chr10 represented ten maize chromosomes; Figure S3. The suggested design of a multi-population GP project. P_a1_, P_a2_, … P_an_ indicate the female parents, P_b1_, P_b2_ … P_bn_ indicate the male parents, n is an integer larger than 2; Figure S4. A diagram illustrating the development of the four hybrid populations; Figure S5. GP schemes across different populations. (a) The within-population scheme; (b) The one-to-one scheme; (c) The three-to-one scheme; TP and VP are training and validation populations, respectively. Table S1. Phenotypic correlation coefficient among yield of each population in the three environments, Table S2. Variance component dissection by using single- and multi-environment yield data, Table S3. Two cross validation schemes—CV1 and CV2. Table S4. PAs of SE, AE and G × E model, Table S5 Genetic information of the parental lines of population 4.
