# Supplementary material for: Genomic Prediction across Structured Hybrid Populations and Environments in Maize
Source: Plants (Basel). 2021 Jun 9;10(6):1174. doi: 10.3390/plants10061174 (PMC8227059; doi:10.3390/plants10061174)
Supplement: Supplementary file 1 [file plants-10-01174-s001.zip › Table S1.pdf]

Table S1 Phenotypic correlation coefficients among yield of each population in three environments

	Population 1		Population 2		Population 3		Population 4	
	XJ	HN	XJ	HN	XJ	HN	XJ	HN
BJ	0.31***	0.32***	0.50***	0.41***	0.36**	0.30*	0.38**	0.37**
XJ		0.41***		0.46***		0.24^{ns}		0.57***

Note: *** indicates $P < 0.001$; ** indicates $P < 0.01$; * indicates $P < 0.05$; ns indicates not significant. BJ, XJ and HN indicate three environments
