# Supplementary material for: Genomic Prediction across Structured Hybrid Populations and Environments in Maize
Source: Plants (Basel). 2021 Jun 9;10(6):1174. doi: 10.3390/plants10061174 (PMC8227059; doi:10.3390/plants10061174)
Supplement: Supplementary file 1 [file plants-10-01174-s001.zip › Table S2.pdf]

Table S2 Variance component dissection by using single- and multi-environment yield data

Environment	σ_g^2	σ_{ge}^2	σ_ε^2	e	r	H^2
BJ	203.51	/	264.02	1	2	0.61
XJ	234.17	/	199.73	1	2	0.70
HN	105.19	/	138.13	1	2	0.60
BJ-XJ-HN	125.20	54.40	203.60	3	2	0.71

Note: σ_g^2 indicates variance of genotype; σ_{ge}^2 indicates variance of genotype-by-environment; σ_ε^2 indicates variance of error; e and r are the number of environments and replicates, respectively; H^2 indicates broad sense heritability; / indicates σ_{ge}^2 can't be estimated using single-environment data
