# Supplementary material for: Genomic Prediction across Structured Hybrid Populations and Environments in Maize
Source: Plants (Basel). 2021 Jun 9;10(6):1174. doi: 10.3390/plants10061174 (PMC8227059; doi:10.3390/plants10061174)
Supplement: Supplementary file 1 [file plants-10-01174-s001.zip › Table S3.pdf]

Table S3 Two cross validation schemes – CV1 and CV2

	CV1		CV2	
	Env1	Env2	Env1	Env2
Line_1	No	No	No	Yes
Line_2	No	No	No	Yes
Line_3	No	No	No	Yes
Line_4	No	No	No	Yes
Line_5	Yes	Yes	Yes	Yes
Line_6	Yes	Yes	Yes	Yes
Line_7	Yes	Yes	Yes	No
Line_8	Yes	Yes	Yes	No
Line_9	Yes	Yes	Yes	No
Line_10	Yes	Yes	Yes	No
Line_11	Yes	Yes	Yes	Yes
Line_12	Yes	Yes	Yes	Yes
Line_13	Yes	Yes	Yes	Yes
…	Yes	Yes	Yes	Yes
Line_n	Yes	Yes	Yes	Yes

Env indicates environment; Line_1 to Line_n indicated different lines of the population; Yes indicates these lines have both genotype and phenotype data; No indicates these lines only have genotype data
