# Supplementary material for: Genomic Prediction across Structured Hybrid Populations and Environments in Maize
Source: Plants (Basel). 2021 Jun 9;10(6):1174. doi: 10.3390/plants10061174 (PMC8227059; doi:10.3390/plants10061174)
Supplement: Supplementary file 1 [file plants-10-01174-s001.zip › Table S4.pdf]

Table S4 PAs of SE, AE and G × E model

CV schemes	Training dataset	validation dataset	SE	AE	G × E
CV1	BJ_XJ	BJ	0.641(0.053)	0.645(0.053)	0.650(0.052)
		XJ	0.736(0.046)	0.727(0.044)	0.737(0.045)
	BJ_ HN	BJ	0.634(0.052)	0.616(0.059)	0.638(0.052)
		HN	0.621(0.064)	0.534(0.076)	0.621(0.067)
	XJ_ HN	XJ	0.739(0.039)	0.721(0.042)	0.744(0.039)
		HN	0.631(0.053)	0.551(0.057)	0.636(0.053)
	BJ_XJ_ HN	BJ	0.630(0.048)	0.624(0.050)	0.639(0.050)
		XJ	0.733(0.040)	0.722(0.043)	0.739(0.040)
		HN	0.622(0.053)	0.522(0.062)	0.621(0.053)
CV2	BJ_XJ	BJ	0.639(0.046)	0.664(0.043)	0.671(0.042)
		XJ	0.739(0.034)	0.747(0.033)	0.758(0.032)
	BJ_ HN	BJ	0.644(0.046)	0.640(0.047)	0.659(0.043)
		HN	0.634(0.050)	0.561(0.054)	0.650(0.048
	XJ_ HN	XJ	0.741(0.069)	0.729(0.09)	0.756(0.062)
		HN	0.631(0.047)	0.574(0.053)	0.663(0.047)
	BJ_XJ_HN	BJ	0.642(0.038)	0.663(0.037)	0.678(0.033)
		XJ	0.739(0.035)	0.753(0.033)	0.769(0.030)
		HN	0.622(0.046)	0.543(0.053)	0.651(0.049)

Note: Training and validation datasets contain YPP data in the indicated environments. For example, in the first row of “Training dataset” column, BJ_XJ indicates that lines in BJ and XJ were used as the training population, and BJ indicates that lines in BJ were used as the validation population. The GP model were run with five-fold CV for 100 repeats, data in the column of “SE”, “AE”, and “G × E” are the mean values of the 100 repeats, and the values in the brackets are the standard deviation of 100 repeats. CV1 and CV2 schemes are shown in Table S3
