# Supplementary material for: Genomic Prediction across Structured Hybrid Populations and Environments in Maize
Source: Plants (Basel). 2021 Jun 9;10(6):1174. doi: 10.3390/plants10061174 (PMC8227059; doi:10.3390/plants10061174)
Supplement: Supplementary file 1 [file plants-10-01174-s001.zip › Table S5.pdf]

Table S5 Genetic information of the parental lines of population 4

Lines of population 4	Generation of parental lines	Sources
pop4_5974	F_3	Hybrid variety[2]
pop4_5893	F_4	Hybrid variety[2]
pop4_5899	F_4	Hybrid variety[2]
pop4_5903	F_4	Hybrid variety[2]
pop4_5907	F_4	Hybrid variety[2]
pop4_5915	F_4	Hybrid variety[2]
pop4_5918	F_4	Hybrid variety[2]
pop4_5925	F_4	Hybrid variety[2]
pop4_5930	F_4	Hybrid variety[2]
pop4_5954	F_4	Hybrid variety[2]
pop4_5958	F_4	Hybrid variety[2]
pop4_5962	F_4	Hybrid variety[2]
pop4_5966	F_4	Hybrid variety[2]
pop4_5978	F_4	Hybrid variety[2]
pop4_5982	F_4	Hybrid variety[2]
pop4_5991	F_4	Hybrid variety[2]
pop4_5994	F_4	Hybrid variety[2]
pop4_5999	F_4	Hybrid variety[2]
pop4_6003	F_4	Hybrid variety[2]
pop4_6014	F_4	Hybrid variety[2]
pop4_5895	F_5	Hybrid variety[2]
pop4_5910	F_5	Hybrid variety[2]
pop4_5922	F_5	Hybrid variety[2]
pop4_5939	F_5	Hybrid variety[2]
pop4_5942	F_5	Hybrid variety[2]
pop4_5946	F_5	Hybrid variety[2]
pop4_5950	F_5	Hybrid variety[2]
pop4_5971	F_5	Hybrid variety[2]
pop4_5986	F_5	Hybrid variety[2]
pop4_6007	F_5	Hybrid variety[2]
pop4_6010	F_5	Hybrid variety[2]
pop4_6019	F_5	Hybrid variety[2]
pop4_6023	F_5	Hybrid variety[2]
pop4_6046	F_5	Hybrid variety[2]
pop4_6055	F_5	Hybrid variety[2]
pop4_6059	F_5	Hybrid variety[2]
pop4_6067	F_5	Hybrid variety[2]
pop4_6071	F_5	Hybrid variety[2]

pop4_6089	F_5	Hybrid variety[2]
pop4_5810	F_n[1]	Hybrid variety[2]
pop4_5814	F_n	Hybrid variety[2]
pop4_5819	F_n	Hybrid variety[2]
pop4_5823	F_n	Hybrid variety[2]
pop4_5827	F_n	Hybrid variety[2]
pop4_5831	F_n	Hybrid variety[2]
pop4_5834	F_n	Hybrid variety[2]
pop4_5838	F_n	Hybrid variety[2]
pop4_5842	F_n	Hybrid variety[2]
pop4_5846	F_n	Hybrid variety[2]
pop4_5850	F_n	Hybrid variety[2]
pop4_5854	F_n	Hybrid variety[2]
pop4_5858	F_n	Hybrid variety[2]
pop4_5862	F_n	Hybrid variety[2]
pop4_5866	F_n	Hybrid variety[2]
pop4_5870	F_n	Hybrid variety[2]
pop4_5874	F_n	Hybrid variety[2]
pop4_5878	F_n	Hybrid variety[2]
pop4_5882	F_n	Hybrid variety[2]
pop4_5886	F_n	Hybrid variety[2]
pop4_5935	F_n	Hybrid variety[2]
pop4_6026	F_n	Hybrid variety[2]
pop4_6031	F_n	Hybrid variety[2]
pop4_6051	F_n	Hybrid variety[2]
pop4_6062	F_n	Hybrid variety[2]
pop4_6074	F_n	Hybrid variety[2]
pop4_6078	F_n	Hybrid variety[2]
pop4_6083	F_n	Hybrid variety[2]
pop4_5806	F_n	Hybrid variety[2]

Note: Note: 1, the subscript n is an integer ≥ 6； 2, the parental lines were selected from different hybrid varieties.
