# Supplementary figures and images for: Genomic Prediction across Structured Hybrid Populations and Environments in Maize
Source: Plants (Basel). 2021 Jun 9;10(6):1174. doi: 10.3390/plants10061174 (PMC8227059; doi:10.3390/plants10061174)

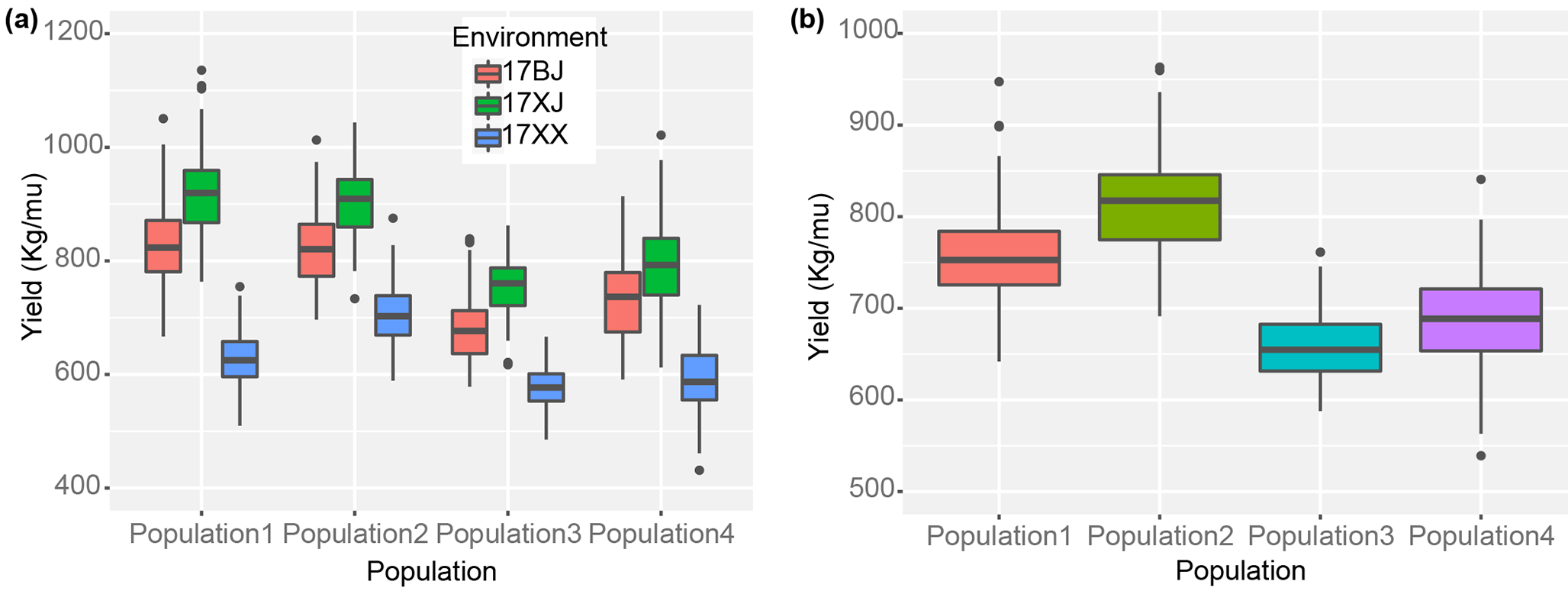

Supplement: Supplementary file 1 [file plants-10-01174-s001.zip › Figure S1.tif]

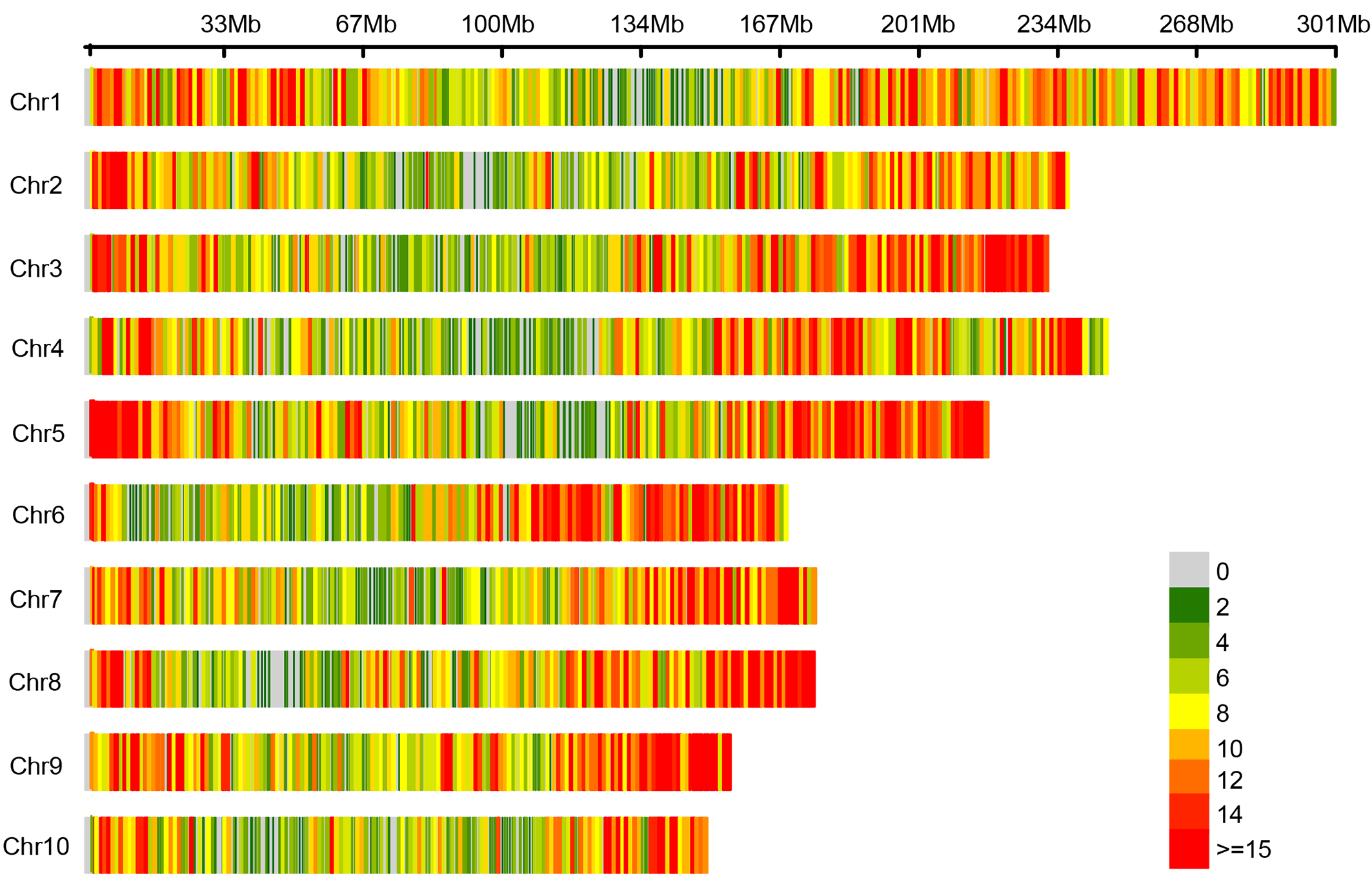

Supplement: Supplementary file 1 [file plants-10-01174-s001.zip › Figure S2.tif]

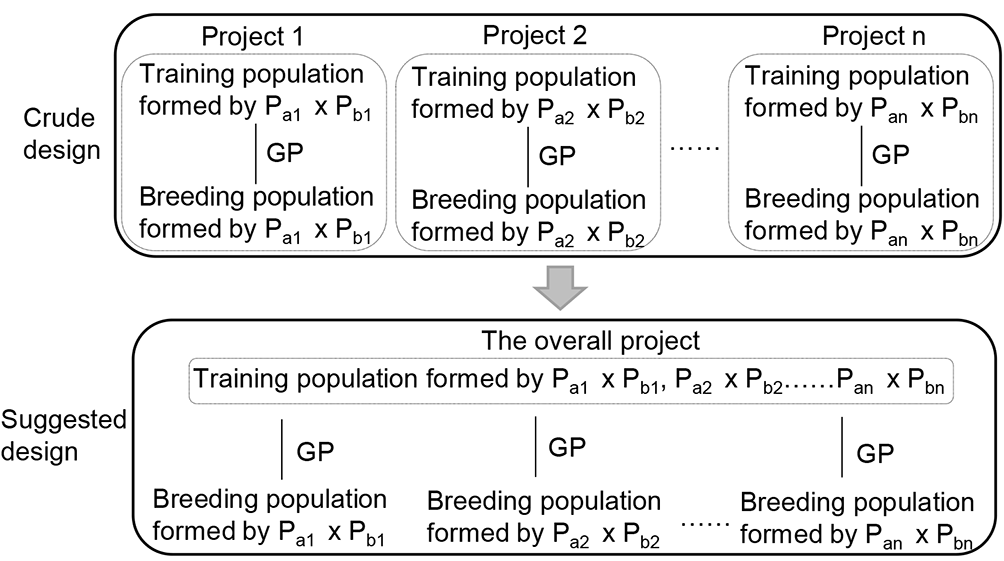

Supplement: Supplementary file 1 [file plants-10-01174-s001.zip › Figure S3.tif]

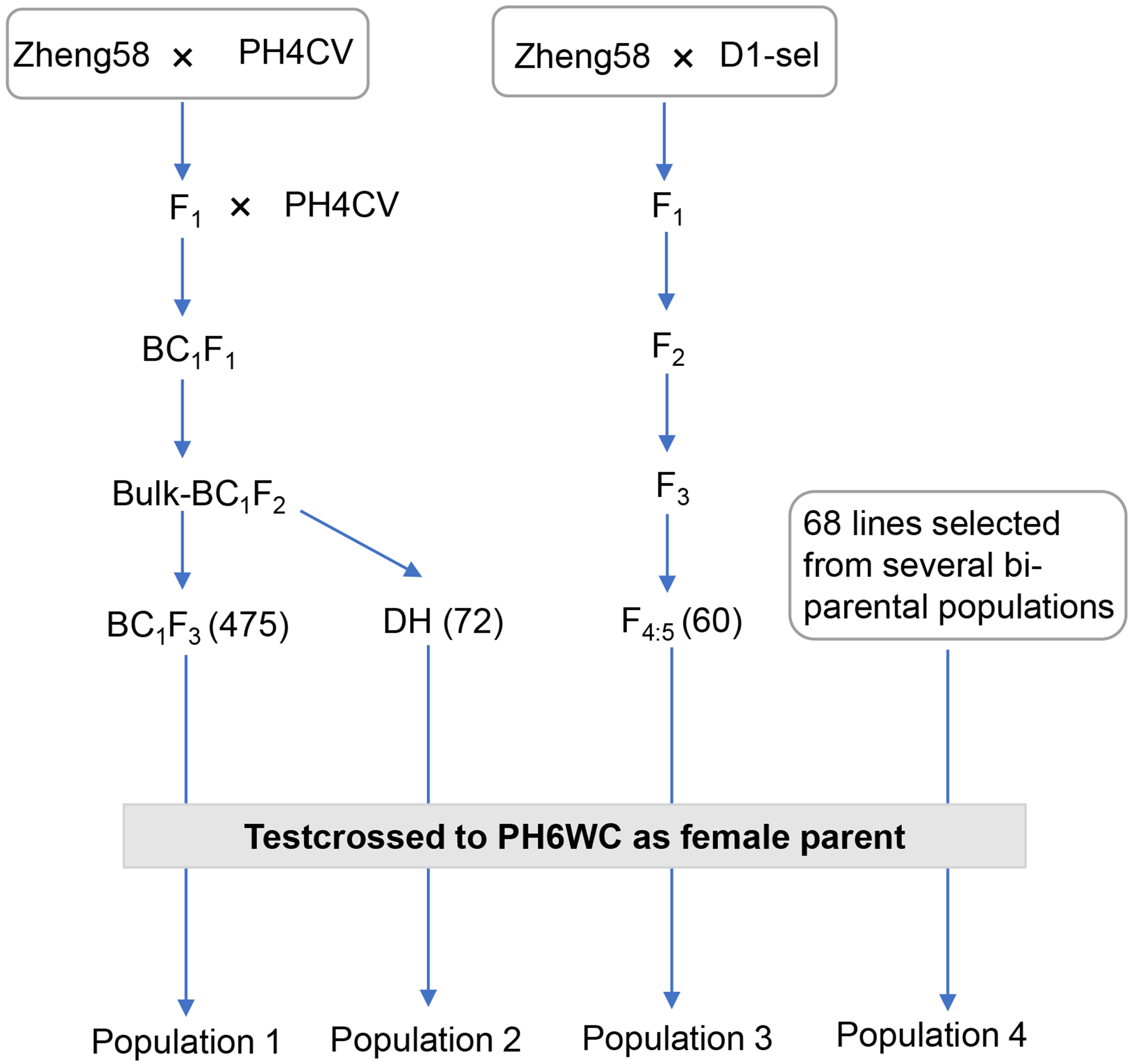

Supplement: Supplementary file 1 [file plants-10-01174-s001.zip › Figure S4.tif]

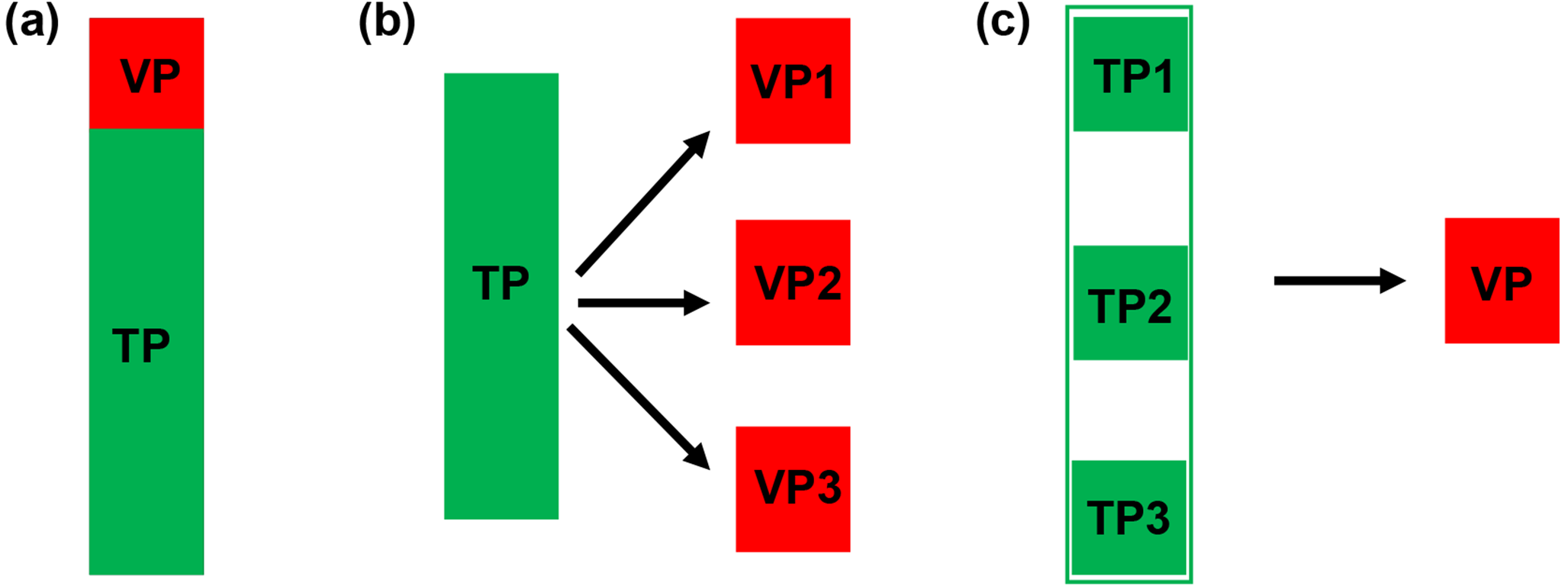

Supplement: Supplementary file 1 [file plants-10-01174-s001.zip › Figure S5.tif]
